# The Australian music industry’s mental health crisis: media narratives during the coronavirus pandemic

**DOI:** 10.1177/1329878X20948957

**Published:** 2021-02

**Authors:** Shelley Brunt, Kat Nelligan

**Affiliations:** RMIT University, Australia

**Keywords:** Australia, Australian music industry, coronavirus, live music, media, media narrative, mental health, music, music industry, popular music

## Abstract

How has the Australian music industry’s mental health crisis played out in the media during the coronavirus pandemic? This commentary article considers a snapshot of media reports about this issue. We survey print and online media, press releases, official websites, online seminars and social media from March to June 2020. During this time, the industry has faced financial loss, job insecurity and anxiety for the future of Australian music, thus placing unprecedented strain on an industry already characterised by poor mental health. We identify four key narratives communicated by the media, which we call (1) acknowledging grief and loss, (2) supporting creativity and well-being, (3) adapting to the new normal and (4) envisaging a post-pandemic future. These narratives illustrate overarching concern for music industry workers’ mental health and also the provision of helpful strategies for managing these issues.

The Australian music industry was the first sector to be severely impacted by the coronavirus. On 13 March 2020, Prime Minister Scott Morrison’s ban on non-essential social gatherings foreshadowed the national closure of live music venues, and the beginning of the end. It was a day when ‘the music died’ along with ‘$100 million [AUD] in wages across the live music industry’ (Donoughue, 2020), later increasing to an estimated $340 million (I Lost My Gig Australia, 2020). The federal government’s insufficient financial bailout added insult to this injury, leaving Australia’s musicians, managers, venue bookers and other industry workers undervalued, despite music’s proven status as an economic generator (Newton and Coyle-Hayward, 2017: 8) and as integral to Australian culture (Victorian Music Development Office (VMDO), 2019: 4). In sum, the pandemic has resulted in financial loss, job insecurity and anxiety for the future of Australian music, thus placing unprecedented strain on an industry already characterised by poor mental health, long working hours and ‘burn out’ (Strong et al., 2020: 46).^1^

Given the multitude of competing reports concerning health, economics and culture that exist in the endless coronavirus news cycle, it is no wonder that the media has failed to prioritise the devastating effects of the pandemic on the mental health of music industry workers. In this short commentary article, we pay critical attention to what *has* been communicated, asking: what media narratives about the Australian music industry’s mental health crisis have emerged during the coronavirus pandemic? As Melbourne-based popular music academics, we are especially mindful of our local industry, given ‘Melbourne has the most live music venues and more live music venues per capita of any global city’ (Newton and Coyle-Hayward, 2017: 6), but we broaden our scope here to consider Australia-wide media – print, online, press releases, official websites, online seminars and social media – to capture a communication snapshot for the period 13 March to 1 June 2020. In doing so, we have noticed four recurring narratives, which we call (1) acknowledging grief and loss, (2) supporting creativity and wellbeing, (3) adapting to the new normal and (4) envisaging a post-pandemic future. It is hoped that this consolidated commentary, which represents our evolving thoughts, can be used as a future resource for studies on mental health, the music industry, media, communication and the coronavirus pandemic.

## Narrative 1: acknowledging grief and loss

The first narrative articulates intensely personal accounts of grief and loss that musicians are experiencing during the coronavirus pandemic, which directly impacts their mental health. Such articles utilise first-person quotes to capture feelings of despair prompted by the forced cancellation of 2020 tours. Music bible, the *NME*, for example, shines a spotlight on songwriter Gordi’s ‘devastating’ and ‘traumatising experience’ of facing no ticket sales, no performance fees and no merchandise sales (Lim, 2020). Musician Ash Grunwald’s grief and loss is tied to financial devastation, pinpointing coronavirus as ‘the biggest challenge of my life’ (Lim, 2020). Industry peak bodies are similarly documenting the hardships musicians face as a result of the live music shutdown. The *Australian Festivals Association* and *Australian Music Industry Network*, for example, rolled out the ‘I Lost My Gig: Australia’ (2020) initiative to tally income and job losses and to capture music industry workers’ personal stories of adversity. Musicians are also bypassing media outlets to directly express their grief and loss via their own social media platforms. Via Instagram, Alex Lahey discusses her cancelled tour and her anxiety in relation to the uncertainty of Melbourne’s live music sector, and she calls on politicians to provide immediate action and financial support (Lahey, 2020). Lahey’s social media post represents but one of many concerned artists’ voices and was circulated by numerous media outlets, including the *ABC* (Newstead, 2020a), *The Brag* (Gray, 2020), *NME* (Young, 2020) and *The Music* (Dale, 2020). Collectively, these intimate stories contribute to an emotive narrative of grief and loss that is expressed across the entire music sector.

## Narrative 2: supporting creativity and wellbeing

Understandably, poor mental health elicits a decline in creativity and productivity for music industry workers. Our informal survey of media sheds light on the strong promotion of coronavirus-related wellbeing resources designed to counter this. Peak bodies for the industry, *Music Victoria* and *Creative Arts Victoria*, actively publicise charity organisation *Support Act*’s financial relief services and 24-hour counselling line to local music industry workers, as well as national mental health services such as *Beyond Blue* and *The Australian Alliance for Wellness in Entertainment*. From March, Support Act’s (2020) live online seminar series ‘Sound Check’ has also offered musicians advice from psychologists and health experts on managing mental health during the pandemic. Music Victoria (2020) has produced one of the most comprehensive support webpages, including ‘Mental Wellbeing Resources’ and ‘Creativity Resources’, with links to external videos, articles, regular meetings, mindfulness content and psychology tips. Such resources represent the new strategies employed by peak bodies to help musicians sustain their creativity and, by corollary, improve their wellbeing during lockdown: a welcome step for advancing wellbeing across the music industry more broadly.

## Narrative 3: adapting to the ‘new normal’

The ongoing ‘new normal’ of lockdown in some places in Australia, or general restrictions in others, has prompted the media to publish a multitude of articles offering professional advice and business survival resources to assist artists with generating income and connecting with fans. This third media narrative goes a step beyond the social imperative of ‘supporting creativity and wellbeing’, which we describe above, and is instead characterised by technical innovation and workarounds to adapt to lockdown, alongside trusted tips from peak bodies. Livestreaming free gigs, band interviews and festivals, such as *Delivered Live, Isol-Aid* and *The State of Music*, are presented as key to maintaining connections with fans. The monetisation of mediatised performances, moreover, is hailed as both a ‘short-term solution’ and a ‘long term movement’ (Newstead, 2020b). Artists use established platforms like Twitch and Patreon alongside emerging technologies, such as StageIt and Veeps, to create new revenue streams during the pandemic. In May, a new Sydney drive-in concert offered an alternative to these online gigs, with one reviewer from The Music prompting readers to #SupportTheBands by donating directly to the artists or to Support Act (Radojkovic, 2020). Importantly, arts organisations such as the VMDO (2020) and Creative Arts Victoria (2020) offer dedicated coronavirus resources links on their websites, providing musicians with information on how to livestream, apply for grants and funding, and release and market music. Time will tell if these workarounds are simply short-term solutions for the ‘new normal’ or if they demonstrate a shift towards new performance trends within the sector more broadly.

## Narrative 4: envisaging a post-pandemic future

The final narrative we have observed displays optimism, resilience and hope, thus signalling a bright but different future for the Australian music industry. These descriptors go hand-in-hand with improvements in wellbeing for industry workers. Many articles indicate that artist managers and industry personnel are confident the industry will bounce back (Cooper, 2020), and artists too note the ‘ever-resourceful and resilient’ traits of the music industry, because it ‘will always find ways to survive’ (Gordi in Lim, 2020). A recent survey conducted by *Live Nation* indicates that regular gig-goers are embodying hope by patiently waiting for the return of live music (Cooper, 2020). Indeed, the potential re-opening of venues in Melbourne, Australia’s live music capital, is positioned by the *ABC* as offering ‘a glimmer of hope’ for live music fans, with ticket prices projected to be higher than in pre-pandemic times to remedy the historic underpayment of artists (Terzon and Knight, 2020). The resilience of musicians amid adversity is also a common theme in music industry papers/websites, as is their unwavering ability to adapt and innovate (Brereton, 2020) – a trait that will suggest success in a post-pandemic world.

## Conclusion

Our four narratives point to the Australian media’s disparate communication of the serious issues facing the music industry during the pandemic. Certainly, there are overarching themes of concern for music industry workers’ mental health, and also the provision of helpful strategies for managing these issues. These are, however, treated by the media as secondary to national concerns about the coronavirus’ impact on the wider Australian economy. As lockdown restrictions slowly ease in some parts of the country, music venues will be one of the last spaces to reopen, with festivals and large-scale concerts remaining ‘low on the [government’s] list of priorities’ (Chief Medical Officer Brendan Murphy in Triscari, 2020). Additional media narratives about the coronavirus and the mental health of Australia’s music industry workers will undoubtedly emerge; as to *what* these narratives will convey remains open to speculation.

## Notes

1. The Australian music industry is an underexplored topic, but in recent years, scholars such as Hughes et al. (2016) have summarised the unique set of challenges musicians must navigate when forging a career. Some of the these directly relate to wellbeing and mental health, and include: financial risk and exploitation (26), being ‘noticed’ or ‘heard’ online (37), intermittent financial remuneration (86), shifting government regulations around live music venues (5), difficulties creating an image and maintaining a brand (89) and broader concerns around workplace health and safety (91). Industry reports also shed light on the mental health conditions of workers. Findings from a 2016 report about the entertainment industries (which includes the music industry) reveals high levels of attempted suicide (more than ‘double that of the general population’), moderate to severe levels of anxiety symptoms ( ‘10 times higher than in the general population’) and high levels of depression (five times higher than in the general population (Entertainment Assist, n.d.). The brevity of our commentary article restricts references to additional publications; however, the two texts named here will serve as a useful starting point for further research.
